# ARSDA: A New Approach for Storing, Transmitting and Analyzing Transcriptomic Data

**DOI:** 10.1534/g3.117.300271

**Published:** 2017-10-27

**Authors:** Xuhua Xia

**Affiliations:** Department of Biology, University of Ottawa, Ontario K1N 6N5, Canada; Ottawa Institute of Systems Biology, Ontario K1H 8M5, Canada

**Keywords:** transcriptomics, novel storage solution, quantifying expression of paralogous genes, sequence format, ARSDA

## Abstract

Two major stumbling blocks exist in high-throughput sequencing (HTS) data analysis. The first is the sheer file size, typically in gigabytes when uncompressed, causing problems in storage, transmission, and analysis. However, these files do not need to be so large, and can be reduced without loss of information. Each HTS file, either in compressed .SRA or plain text .fastq format, contains numerous identical reads stored as separate entries. For example, among 44,603,541 forward reads in the SRR4011234.sra file (from a *Bacillus subtilis* transcriptomic study) deposited at NCBI’s SRA database, one read has 497,027 identical copies. Instead of storing them as separate entries, one can and should store them as a single entry with the SeqID_NumCopy format (which I dub as FASTA+ format). The second is the proper allocation of reads that map equally well to paralogous genes. I illustrate in detail a new method for such allocation. I have developed ARSDA software that implement these new approaches. A number of HTS files for model species are in the process of being processed and deposited at http://coevol.rdc.uottawa.ca to demonstrate that this approach not only saves a huge amount of storage space and transmission bandwidth, but also dramatically reduces time in downstream data analysis. Instead of matching the 497,027 identical reads separately against the *B. subtilis* genome, one only needs to match it once. ARSDA includes functions to take advantage of HTS data in the new sequence format for downstream data analysis such as gene expression characterization. I contrasted gene expression results between ARSDA and Cufflinks so readers can better appreciate the strength of ARSDA. ARSDA is freely available for Windows, Linux. and Macintosh computers at http://dambe.bio.uottawa.ca/ARSDA/ARSDA.aspx.

High-throughput sequencing (HTS) is now used not only in characterizing differential gene expression, but also in many other fields, where it replaces the traditional microarray approach. Ribosomal profiling, traditionally done through microarray (Arava *et al.* 2003; MacKay *et al.* 2004), is now almost exclusively done with deep sequencing of ribosome-protected segments of messages (Ingolia *et al.* 2009, 2011), although the results from the two approaches for ribosomal profiling are largely concordant (Xia *et al.* 2011). Similarly, EST-based (Rogers *et al.* 2012) and microarray-based (Pleiss *et al.* 2007) methods for detecting alternative splicing events and characterizing splicing efficiency is now replaced by HTS (Kawashima *et al.* 2014), especially by lariat sequencing (Awan *et al.* 2013; Stepankiw *et al.* 2015). The availability of HTS data has dramatically accelerated the test of biological hypotheses. For example, a recent study on alternative splicing (Vlasschaert *et al.* 2016) showed that skipping of exon 7 (E_7_) in human and mouse *USP4* is associated with weak signals of splice sites flanking E_7_. The researchers predicted that, in some species where the splice site signals are strong, E_7_ skipping would disappear. This prediction is readily tested and confirmed with existing HTS data, *i.e.*, E_6_-E_8_ mRNA was found in species with weak splice signals flanking E_7_, and E_6_-E_7_-E_8_ mRNA in species with strong splice signals flanking E_7_ (Vlasschaert *et al.* 2016).

In spite of the potential of HTS data in solving practical biological problems, severe underusage of HTS data has been reported (GB Editorial Team 2011). One major stumbling block in using the HTS data are the large file size. Among the 6472 HTS studies on human available at NCBI/DDBJ/EBI by April 14, 2016, 196 studies each contribute >1 Terabyte (TB) of nucleotide bases, with the top one contributing 86.4 TB. Few laboratories would be keen on downloading and analyzing this 86.4 TB of nucleotides, not to mention comparing this study to HTS data from other human HTS studies.

The explosive growth of HTS data in recent years has caused serious problems in data storage, transmission, and analysis (Leinonen *et al.* 2011; Kodama *et al.* 2012). Because of the high cost of maintaining such data, coupled with the fact that few researchers had been using such data, NCBI had planned the closure of the sequence read archive a few years ago (GB Editorial Team 2011), but continued the support only after DDBJ and EBI decided to continue their effort of archiving the data. The incident highlights the prohibitive task of storing, transmitting, and analyzing HTS data, and motivated the joint effort of both industry and academics to search for data compression solutions (Janin *et al.* 2014; Zhu *et al.* 2015b; Numanagic *et al.* 2016).

HTS data files do not need to be so huge. Take, for example, the characterized transcriptomic data for *Escherichia coli* K12 in the file SRR1536586.sra (where SRR1536586 is the SRA sequence file ID in NCBI/DDBJ/EBI). The file contains 6,503,557 sequence reads of 50 nt each, but 195,310 sequences are all identical (TGTTATCACGGGAGACACACGGCGGGTGCTAACGTCCGTCGTGAAGAGGG), all mapping exactly to sites 929–978 in *E. coli* 23S rRNA genes (the study did use the MICROBExpress Bacterial mRNA Enrichment Kit to remove the 16S and 23S rRNA, otherwise there would be many more). There are much more extreme cases. For example, one of the 12 HTS files from a transcriptomic study of *E. coli* (SRR922264.sra), contains a read with 1,606,515 identical copies among its 9,690,570 forward reads. There is no sequence information lost if all these 1,606,515 identical reads are stored by a single sequence with a sequence ID such as UniqueSeqX_1606515 (*i.e.*, SeqID_CopyNumber format which I dub FASTA+ format with file type .fasP). Such storage scheme not only results in dramatic saving in data storage and transmission, but also leads to dramatic reduction in computation time in downstream data analysis. At present, all software packages for HTS data analysis will take the 1,606,515 identical reads separately, and search them individually against the *E. coli* genome (or target gene sequences such as coding sequences). The SeqID_CopyNumber storage scheme reduces the 1,606,515 searches to a single one.

A huge chunk of SRA data stored in NCBI/DDBJ/EBI consists of ribosome profiling data (Ingolia *et al.* 2009, 2011), which is obtained by sequencing the mRNA segment (∼30 bases) protected by the ribosome after digesting all the unprotected mRNA segments. Mapping these ribosome-protected segments to the genome allows one to know specifically where the ribosomes are located along individual mRNAs. In general, such data are essential to understand translation initiation, elongation, and termination efficiencies. For example, a short poly(A) segment with about eight or nine consecutive A immediately upstream of the start codon in yeast (*Saccharomyces cerevisiae*) genes is significantly associated with ribosome density and occupancy (Xia *et al.* 2011), confirming the hypothesis that short poly(A) upstream of the start codon facilitates the recruitment of translation initiation factors, but long poly(A) would bind to poly(A)-binding protein and interfere with cap-dependent translation. Sequence redundancy is high in such ribosomal profiling data and the FASTA+ format can lead to dramatic saving in the disk space of data storage and time in data transmission.

Aside from the file size problem, HST data analysis also suffers from the problem of how to allocate multiple-matched reads to paralogous genes (Trapnell *et al.* 2013; Rogozin *et al.* 2014). The commonly used options of ignoring such multiple-matched reads or allocating them equally among matched paralogous genes are unsatisfactory. The software ARSDA I present here offers solutions to both the problem of file size and the problem of read allocation to paralogous genes.

## ARSDA

**Figure 1:**
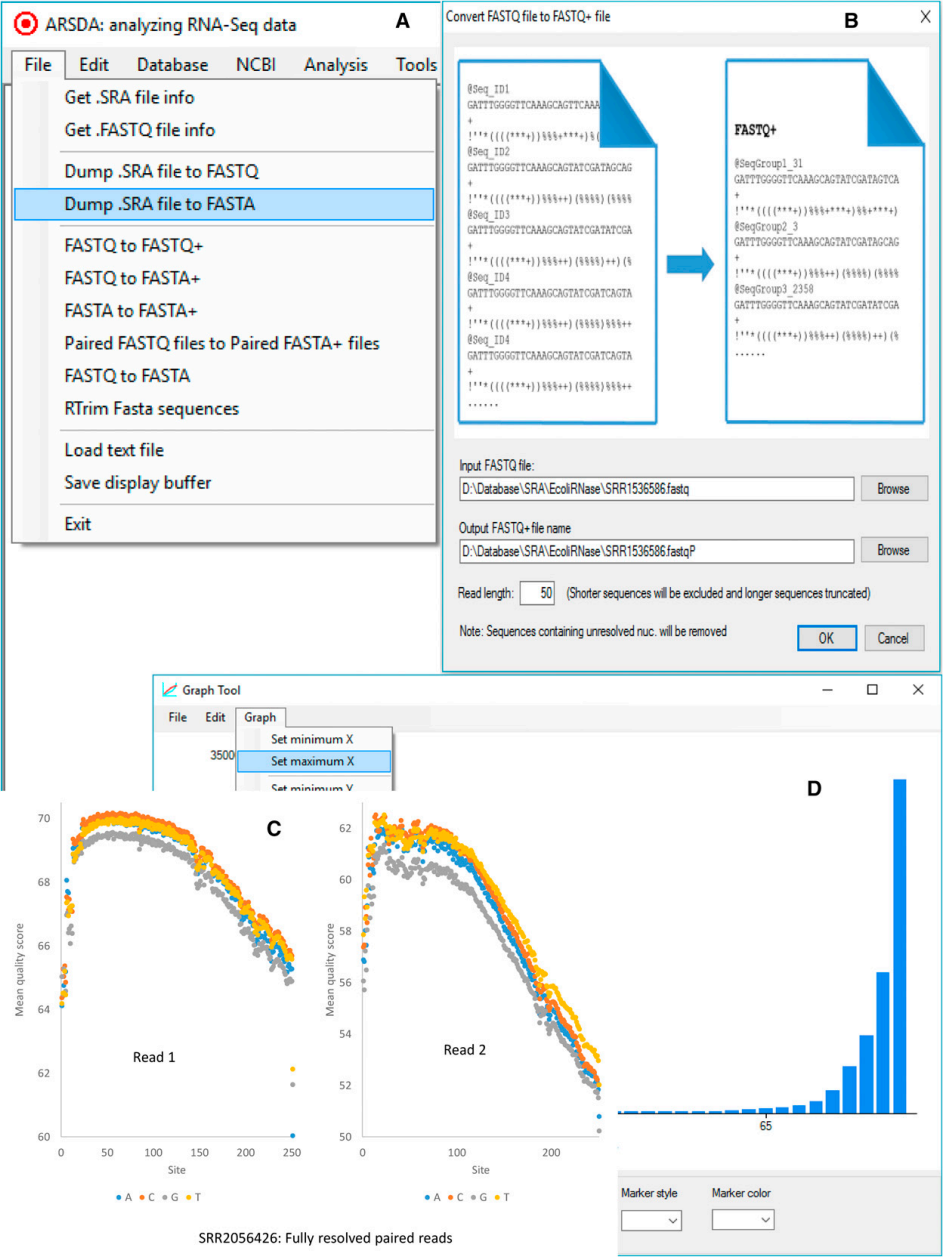
User interface in ARSDA. (A) The menu system, with database creation under the “Database” menu, gene expression characterization under the “Analysis” menu, etc. (B) Converting a FASTQ/FASTA file to a FASTQ+/FASTA+ file. (C) Site-specific read quality visualization. (D) Global read quality visualization.

I developed software ARSDA (for Analyzing RNA-Seq Data, Figure 1A) to alleviate the problem associated with storage, transmission and analysis of HTS data. ARSDA can take input .SRA files or .fastq files of many gigabytes, build an efficient dictionary of unique sequence reads in a FASTA/FASTQ file, keep track of their copy numbers, and output them to a FASTA+ file in the SeqID_CopyNumber format (Figure 1B). Both fixed-length and variable-length sequences can be used as input. In addition, I have implemented functions in ARSDA to take advantage of the new sequence format to perform gene expression, with the main objective of demonstrating how much faster downstream data analysis can be done with data in FASTA+/FASTQ+ format. Furthermore, ARSDA includes a unique feature in assigning shared reads among paralogous genes that I will describe below. ARSDA also includes sequence visualization functions for global base-calling quality, per-read quality, and site-specific read quality (Figure 1, C and D), but these functions are also available elsewhere, *e.g.*, FastQC (Andrews 2017) and NGSQC (Dai *et al.* 2010) and consequently will not be described further (but see the attached QuickStart.PDF). ARSDA includes nine programs in the BLAST and sratoolkit from NCBI that enhance part of ARSDA functions.

### Converting HTS data to FASTA+/FASTQ+

The output from processing the SRR1536586.sra file (with part of the read matching output in Table 1) highlights two points. First, many sequences in the file are identical. Second, although the transcriptomic data characterized in SRR1536586 have undergone rRNA depletion by using Ambion’s MICROBExpress Bacterial mRNA Enrichment Kit (Pobre and Arraiano 2015), there are still numerous reads in the transcriptomic data that are from rRNA genes. This suggests that storing mRNA reads separately from rRNA reads can dramatically reduce file size because most researchers are not interested in the abundance of rRNAs.

**Table 1:** Part of read-matching output from ARSDA, with 195,310 identical reads matching a segment of large subunit (LSU) rRNA, 86,308 identical reads matching another segment of LSU rRNA, and so on

Gene	N_copy_	Gene	N_copy_
LSU rRNA	195,310	SSU rRNA	30,417
LSU rRNA	86,308	LSU rRNA	29,508
LSU rRNA	58,400	5S rRNA	28,187
SSU rRNA	47,323	LSU rRNA	24,982
LSU rRNA	45,695	SSU rRNA	23,286
LSU rRNA	36,258	LSU rRNA	19,991
5S rRNA	33,674	SSU rRNA	19,268

Results generated from ARSDA analysis of the SRR1536586.sra file from NCBI.

While the conversion of FASTA/FASTQ files to FASTA+ files may take a few minutes, it needs to be done only once for data storage, and the resulting saving in storage space, internet traffic, and computation time in downstream data analysis is tremendous. The file size is 1.49 GB for the original FASTQ file derived from SRR1536586.sra, but is only 66 MB for the new FASTA+ file, both being plain text files.

I have applied ARSDA to reduce the file size of transcriptomic data from yeast (*S. cerevisiae*), nematode (*Caenorhabditis elegans*), fruit fly (*Drosophila melanogaster*), zebrafish (*Danio rerio*), and mouse (*Mus musculus*), and deposited the resulting reduced data at coevol.rdc.uottawa.ca in the form of BLAST databases. BLAST reduces sequences further by representing tetranucleotides AAAA, AAAC, ..., TTTT by 0, 1, ..., 255 so that each tetranucleotide takes only 1 byte in storage. The sequence ID in these BLAST databases are in the form of SeqID_CopyNumber. These files reduce the computation time for quantifying gene expression from many hours to only a few minutes (>2 min for my Windows 10 PC with an i7-4770 CPU at 3.4 GHz and 16 GB of RAM). This eliminates one of the key bottlenecks in HTS data analysis (Liu *et al.* 2016), and would make it feasible for any laboratory to gain the power of HTS data analysis. I attach the gene expression characterized by ARSDA for the 4321 *E. coli* K12 coding sequences as Supplemental Material, File S1. A part of it is reproduced in Table 2.

**Table 2:** Partial output of gene expression, with the gene locus_tag (together with start and end sites) as Gene ID

Gene ID	SeqLen	Count	Count/Kb	FPKM
b0001|190_255	66	76	1151.515	389.894
b0002|337_2799	2463	2963	1203.004	407.328
b0003|2801_3733	933	1121	1201.501	406.819
b0004|3734_5020	1287	1782	1384.615	468.82
b0005|5234_5530	297	97	326.599	110.584
b0006|C5683_6459	777	113	145.431	49.242
b0007|C6529_7959	1431	143	99.93	33.836
b0008|8238_9191	954	1561	1636.268	554.028
b0009|9306_9893	588	289	491.497	166.417
b0010|C9928_10494	567	100	176.367	59.716
b0011|C10643_11356	714	13	18.207	6.165
b0013|C11382_11786	405	2	4.938	1.672
b0014|12163_14079	1917	6863	3580.073	1212.186
b0015|14168_15298	1131	1671	1477.454	500.255
…	…	…	…	…

One may ask how quality scores are treated when reads with identical sequences are grouped into the form of SeqID_CopyNumber format. Let me first highlight two observations. First, different reads with the same sequence have similar quality scores. For example, sequence “TGTTATCACGGGAGACACACGGCGGGTGCTAACGTCCGTCGTGAAGAGGG” occur many times in file SRR1526586.sra. I took the first six reads with this 50-nt sequence, and computed Pearson correlation among the associated quality scores (each read is associated with a vector of 50 quality scores). The correlation coefficients are all high and positive (Table 3). The same for sequences that occur just twice. Second, quality score itself is a statistical estimate. For these reasons, when reads with the same sequence are combined into the SeqID_CopyNumber format in Fastq+ file, the quality scores for this combined sequence are simply average quality scores. For a sequence of length *L* that occurred twice in the transcriptomic data, the sequence ID will be SeqID_2, and quality scores will simply be (Q_1i_ + Q_2i_)/2, where *i* = 1, 2, …, *L*, and Q1 and Q2 are quality scores from the two reads of the same sequence.

**Table 3:** Correlation among quality scores from first six reads (Q1–Q6) of the same sequence of 50 nt (TGTTATCACGGGAGACACACGGCGGGTGCTAACGTCCGTCGTGAAGAGGG)

	Q1	Q2	Q3	Q4	Q5
Q2	0.804889				
Q3	0.662242	0.873874			
Q4	0.71594	0.938951	0.918316		
Q5	0.784977	0.968069	0.864678	0.945969	
Q6	0.634808	0.850372	0.926804	0.931704	0.866401

Each read is associated with a vector of 50 quality scores (one for each nucleotide).

**Figure 2:**
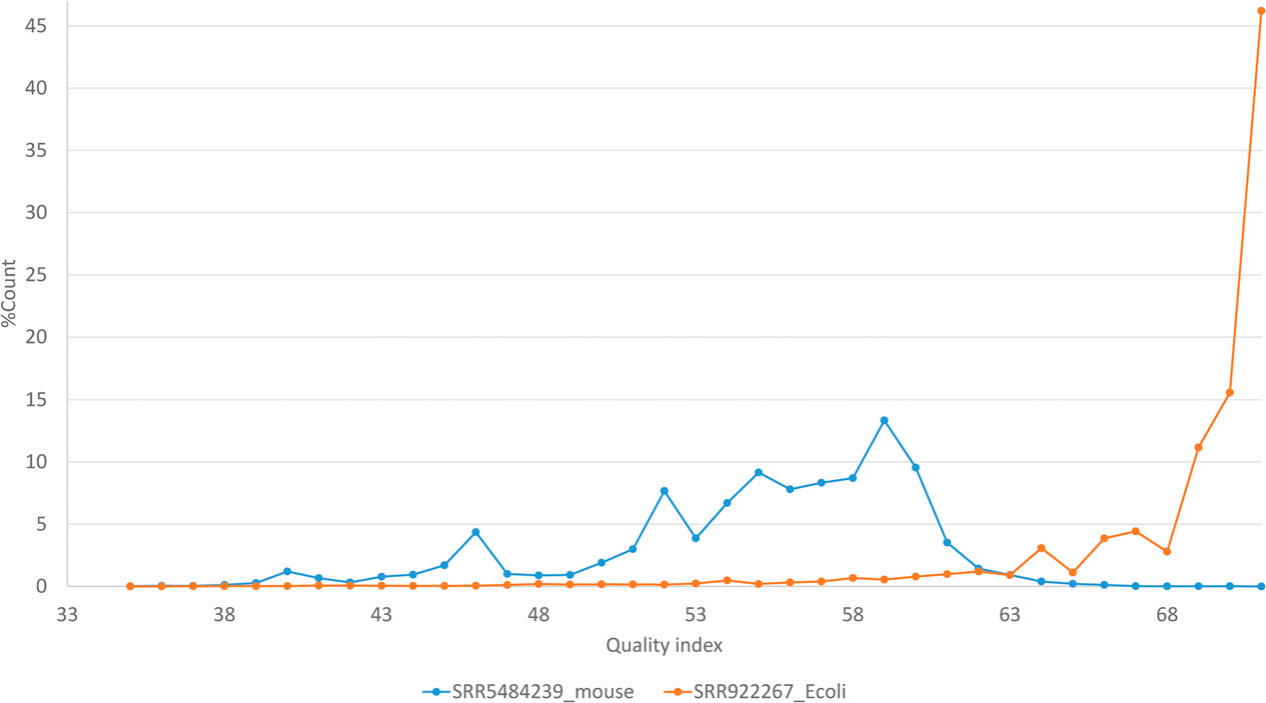
Contrasting read quality between two transcriptomic data files (SRR5484239.sra from *M. musculus* and SRR922267.sra from *E. coli*. It does not imply that *E. coli* data are always better than mouse data as there are also poor-quality *E. coli* data and high-quality mouse data.

Size-reduction differs dramatically with read quality (Figure 2). For high-quality data, *e.g.*, SRR922267 (Figure 2, where an overwhelming majority of bases are at the high-quality end), ARSDA can shrink file size to ∼0.05 of the original. However, for poor-quality data, *e.g.*, SRR5484239 (Figure 2), ARSDA can shrink file size only to 0.64 of the original. The reason is that, with high-quality data, reads from the same segment of the transcript are identical, as one would expect, but, with low-quality data, reads from the same segment of the transcript have “mutated” during the amplification and sequencing step, and are often no longer identical. For SRR922267, the most redundant sequence has 2,341,386 identical copies out of 14,872,404 forward reads. In contrast, the most redundant sequence in SRR5484239 has only 1540 identical copies out of 10,702,871 reads. This implies that the paired-end reads, especially long ones, will likely have low size-reduction efficiency because reverse read quality is typically much worse than forward read quality. Size-reduction with the ARSDA approach works best with high-quality reads. Base-calling quality typically decreases rapidly with read length (Figure 1C). Trimming off the low-quality 3′ end of the reads typically leads to dramatically increased size-reduction efficiency.

One of the frequently used sequence-compression scheme is to use a reference genome so that each read can be represented by a starting and an ending number on the genome (Benoit *et al.* 2015; Kingsford and Patro 2015; Zhu *et al.* 2015a). This approach has two problems. First, many reads do not map exactly to the genomic sequence because of either somatic mutations or sequencing errors, so representing a read by the starting and ending numbers leads to loss of information. Second, RNA-editing and processing can be so extensive that it becomes impossible to map a transcriptomic read to the genome (Abraham *et al.* 1988; Lamond 1988; Alatortsev *et al.* 2008; Li *et al.* 2009; Simpson *et al.* 2016). Furthermore, there are still many scientifically interesting species that do not have a good genomic sequence available. One could add additional annotation and indexing for sequence variants resulting from RNA-editing and “mutants” resulting from amplification and sequencing to avoid information loss, but such additional steps reduces the efficiency of compression as well as increases an extra layer of complexity for downstream data analysis.

Software tools for compressing HTS files are often benchmarked against general-purpose GZIP tools (Numanagic *et al.* 2016). Among nonreference compression tools for FASTQ files, LFQC (Nicolae *et al.* 2015) was benchmarked to be the most efficient (Numanagic *et al.* 2016), partly because LFQC uses several compression programs separately for sequence IDs, sequences, and quality scores. I compared file size reduction from FASTQ+ format against compression results from GZIP and LFQC (Table 4). Because FASTQ+ files are simple text files that can be further compressed by GZIP and LFQC, Table 4 also include compression output of GZIP+FASTQ+ and LFQC+FASTQ+. The results (Table 4) confirms the previous finding (Numanagic *et al.* 2016) that LFQC is much more efficient than GZIP. They also show FASTQ+ to offer a substantial further reduction of file sizes. For SRR1536586, file size reduction efficiency is comparable between LFQC and FASTQ+ (Table 4). However, further compression with GZIP+FASTQ+ and LFQC+FASTQ+ both leads to much reduced file size than using GZIP or LFQC alone (Table 4), the same being true for the paired-end file SRR922270 (Table 4). Furthermore, FASTQ+ has one additional advantage in that it dramatically reduces computation time in downstream data analysis. Take SRR1536586 for example, FASTQ+ would reduce computation time for read-matching (which is the most time-consuming part of any transcriptomic data analysis) to a fraction of roughly 0.075 (≈119,596,093/1,604,183,348).

**Table 4:** Comparison of different compression methods from the original single-read file (SRR1536586) and paired-end read file (SRR922270) in FASTQ format

	File Size (in Bytes)		
Methods	SRR1536586	SRR922270_1	SRR922270_2
FASTQ	1,604,183,348	2,647,494,360	2,647,494,360
GZIP	299,347,123	441,010,173	466,626,719
LFQC	101,191,680	159,732,224	174,810,624
FASTQ+*^a^*	119,596,093	493,950,425	493,950,425
GZIP+FASTQ+	35,078,888	130,605,692	130,546,296
LFQC+FASTQ+	16,506,880	61,696,000	62,689,280

SRR1536586 and SRR922270 are SRA file IDs in NCBI SRA database.

a After converting FASTAQ+ format, the quality score for an entry such as SeqID_200 is the mean for the 200 reads and not for individual sequences.

### Assigning sequence reads to paralogous genes

One of the most fundamental objectives of RNA-Seq analysis is to generate an index of gene expression (FPKM: matched fragment/reads per kilobases of transcript per million mapped reads) that can be directly compared among different genes and among different experiments with different total number of matched reads (Mortazavi *et al.* 2008). The main difficulty in quantifying gene expression arises with sequence reads matching multiple paralogous genes (Trapnell *et al.* 2013; Rogozin *et al.* 2014). This problem, which has plagued microarray data analysis, is now plaguing RNA-Seq analysis. Most publications of commonly used RNA-Seq analysis methods (Langmead *et al.* 2009, 2010; Trapnell *et al.* 2009, 2012; Roberts *et al.* 2011, 2013; Langmead and Salzberg 2012; Dobin *et al.* 2013; Deng *et al.* 2014) often avoided mentioning read allocation to paralogous genes. The software tools associated with these publications share two simple options for handling matches to paralogous genes. The first is to use only uniquely matched reads, *i.e.*, reads that match to multiple genes are simply ignored. The second is to assign such reads equally among matched genes. These options are obviously unsatisfactory. Here, I describe a new approach which should substantially improve the accuracy of HTS data analysis such as gene expression characterization.

### Allocating sequence reads to paralogous genes in a two-member gene family

We need a few definitions to explain the allocation. Let *L*_1_ and *L*_2_ be the sequence length of the two paralogous genes. Let *N_U_*_.1_ and *N_U_*_.2_ be the number of reads that can be uniquely assigned to paralogous gene 1 or 2, respectively (*i.e.*, the reads that matches one gene better than the other). Now for those reads that match the two genes equally well, the proportion of the reads contributed by paralogous gene 1 may be simply estimated as

$$P_{1}=\frac{N_{U.1}}{N_{U.1}+N_{U.2}}$$ (1)

Now, for any read that matches the two paralogous genes equally well, we will assign *P*_1_ to paralogous gene 1, and (1−*P*_1_) to paralogous gene 2. In the extreme case when paralogous genes are all identical, then *N_U_*_.1_ = *N_U_*_.2_ = 0, and we will assign 1/2 of these equally matched read to genes 1 and 2. We should modify Equation (1) to make it more generally applicable as follows

$$P_{1}=\frac{0.01+N_{U.1}}{0.02+N_{U.1}+N_{U.2}}$$ (2)

where 0.01 in the numerator and 0.02 in the denominator are pseudocounts. The treatment in Equation (2) implies that, when *N_U_*_.1_ = *N_U_*_.2_ = 0(*e.g.*, when two paralogous genes are perfectly identical), then a read matching equally well to these paralogous genes will be equally divided among the two paralogues.

One problem with this treatment is its assumption of *L*_1_ = *L*_2_. If paralogous gene 1 is much longer than the other, then *N_U_*_.1_ is expected to be larger than *N_U_*_.2_, everything else being equal. One may standardize *N_U_*_.1_ and *N_U_*_.2_ to number of unique matches per 1000 nt, designated by *SN_U.i_* = 1000*N_U.i_*/*L*_i_ (where *i* = 1 or 2) and replace *N_U.i_* in Equation (2) by *SN_U.i_* as follows (Mortazavi *et al.* 2008):

$$P_{1}=\frac{0.01+SN_{U.1}\;}{0.02+SN_{U.1}+SN_{U.2}}=\frac{0.01+\frac{1000N_{U.1}}{L_{1}}}{0.02+1000\left(\frac{N_{U.1}}{L_{1}}+\frac{N_{U.2}}{L_{2}}\right)}$$ (3)

### Allocating sequence reads in gene family with more than two members

One might, mistakenly, think that it is quite simple to extend Equation (3) for a gene family of two members to a gene family with *F* members by writing

$$P_{i}=\frac{0.01+\frac{1000N_{U.i}}{L_{i}}}{0.01F+1000\sum_{i=1}^{F}\frac{N_{U.i}}{L_{i}}}$$ (4)

This does not work. For example, if we have three paralogous genes designated A, B, and C, respectively. Suppose that the gene duplication that gave rise to B and C occurred very recently so that B and C are identical, but A and the ancestor of B and C have diverged for a long time. In this case, *N_U.B_* = *N_U.C_* = 0 because a read matching B will always match C equally well, but *N_U.A_* may be >0. This will result in unfair allocation of many transcripts from B and C to A according to Equation (4). I outline the approach below for dealing with gene families with more than two members.

**Figure 3:**
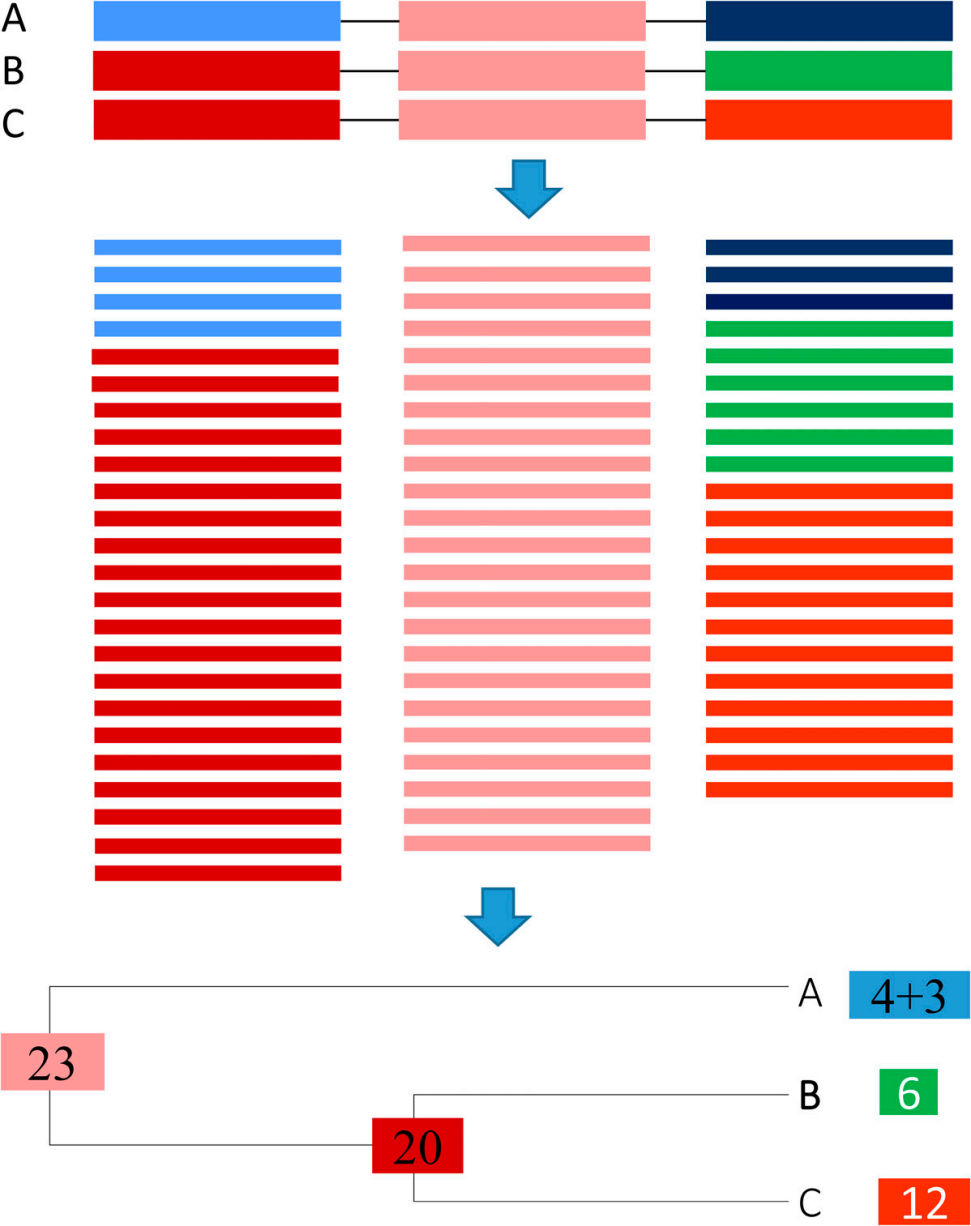
Allocation of shared reads in a gene family with three paralogous genes A, B, and C with three idealized segments with a conserved identical middle segment, strongly homologous first segment that is identical in B and C, and a diverged third segment. Reads and the gene segment they match to are of the same color.

With three or more paralogous genes, one may benefit from a phylogenetic tree for proper allocation of sequence reads. I illustrate the simplest case with a gene family with three paralogous genes A, B, and C, idealized into three segments in Figure 3. The three genes shared one identical middle segment with 23 matched reads (that necessarily match equally well to all three paralogues). Genes B and C share an identical first segment to which 20 reads matched. Gene A has its first segment different from that of B and C, and got four matched reads. The three genes also have a diverged third segment where A matched three reads, B matched six and C matched 12. Our task is then to allocate the 23 reads shared by all three, and 20 reads shared by B and C to the three paralogues.

One could apply maximum likelihood or least-squares method for the estimation, but ARSDA uses a simple counting approach by applying the following

$$\begin{array}{l} P_{A}=\frac{3+4}{3+4+20+6+12}=0.15556\\ P_{B}\;=\;\left(1-P_{A}\right)\frac{6}{6+12}=0.28148\\ P_{C}\;=\;\left(1-P_{A}\right)\frac{12}{6+12}=0.56296 \end{array}$$ (5)

Thus, we allocate the 23 reads (that matched three genes equally) to paralogous genes A, B, and C according to *P_A_*, *P_B_*, and *P_C_*, respectively. For the 20 reads that matched B and C equally well, we allocate 20*6/(6 + 12) to B and 20*12/(6 + 12) to C. This gives the estimated number of matches to each gene as

$$\begin{array}{l} N_{A}=3+4+23P_{A}=10.57778\\ N_{B}=6+23P_{B}+20\left(\frac{6}{6+12}\right)=19.14074\\ N_{C}=12+23P_{C}+20\left(\frac{12}{6+12}\right)=38.28148 \end{array}$$ (6)

These numbers are then normalized to give FPKM (Mortazavi *et al.* 2008). The current version of ARSDA assume that gene families with more than two members to have roughly the same sequence lengths. This is generally fine with prokaryotes but may become problematic with eukaryotes.

In practice, one can obtain the same results without actually undertaking the extremely slow process of building trees for paralogous genes. One first goes through reads shared by two paralogous genes (*e.g.*, the 20 reads shared by genes B and C in Figure 3) and allocate the reads according to *P_B_* = 6/(6 + 12) = 1/3 and *P_C_* = 12/(6 + 12) = 2/3. Now genes B and C will have 12.66667 (= 6 + 20*P_B_) and 25.33333 (= 12 + 20*P_C_) assigned reads, *i.e.*, *N_U.B_* = 12.66667 and *N_U.C_* = 25.33333. Once we have done with reads shared by two paralogous genes, we go through reads shared by three paralogous genes, *e.g.*, the 23 reads shared by genes A, B, and C in Figure 3. With *N_U.A_* = 7, *N_U.B_* = 12.66667, *N_U.C_* = 25.333333, and *N* = *N_U.A_* + *N_U.B_* + *N_U.C_* = 45, so we have

$$P_{A}=\frac{N_{U.A}}{N}=0.15556;P_{B}=\frac{N_{U.B}}{N}=0.28148;P_{C}=\frac{N_{U.C}}{N}=0.56296$$ (7)

$$\begin{array}{l} N_{A}=7+23P_{A}=10.57778\\ N_{B}=12.66667+23P_{B}=19.14074\\ N_{C}=25.33333+23P_{C}=38.28148, \end{array}$$ (8)

which are the same as shown in Equation (6). This progressive process continues until we have allocated reads shared by the largest number of paralogous genes. The gene expression output in File S1 was obtained in this way.

There are alternative approaches for read allocation among paralogous genes. ARSDA also has an alternative allocation scheme based on BitScores and *e*-values. For example, when a read exhibits strong homology to *N* paralogous genes, but with different *e*-values or BitScores, I will not assign the read to the paralogous gene with the smallest *e*-value (or largest BitScore). Instead, all *N* paralogous genes will get a share of the read according to sequence similarity reflected in *e*-value and BitScore. The simplest scheme based on BitScore is to allocate such a read to paralogous gene *i* according to

$$P_{i}=\frac{BitScore_{i}}{\sum BitScore_{i}},$$ (9)

which would give a paralogous gene with high BitScore a higher share. An alternative based on *e*-value is

$$P_{i}=\frac{K\min\left(E\right)}{E_{i}},$$ (10)

where *E* is *e*-value and *K* is a scaling factor to ensure that ∑*p_i_* = 1. Equation (10) allocates shared reads more to the paralogous gene with a small *e*-value than those with large *e*-value. In practice, Equation (9) is often close to equal allocation, whereas Equation (10) results in more biased allocation favoring the best-match paralogous genes.

## Contrast Between ARSDA and Cufflinks in Characterizing Gene Expression

**Figure 4:**
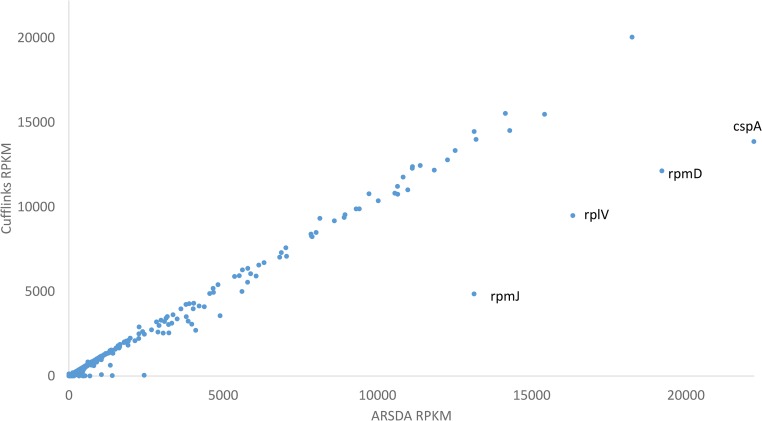
Contrast in gene expression (RPKM) between ARSDA and Cufflinks output for the same transcriptomic data in file SRR1536586.sra for *E. coli* wild type.

The most frequently used software for gene expression is Cufflinks (Trapnell *et al.* 2012), which is why I am contrasting ARSDA against it. I will use the transcriptomic data for an *E. coli* wild type (Pobre and Arraiano 2015), archived in NCBI’s SRA database as SRR1536586.sra. The Cufflinks-quantified gene expression for this file is in file GSM1465035_WT.txt.gz from NCBI Geo DataSets GSM1465035. Gene expression from ARSDA and Cufflinks in mostly concordant (Figure 4), but four points (labeled in Figure 4) stand out as outliers (although many more discordant points will be revealed by a log-log plot). Such dramatic differences demand an explanation. Take *rpmJ* for example. Either ARSDA severely overestimated, or Cufflinks severely underestimated, the gene expression (Figure 4). I originally expected the discrepancy to be due to different allocation of paralogous genes. The expectation is only partially true.

There are 6426 reads can be mapped unambiguously to *rpmJ* (which is in fact a single-copy ribosomal protein gene). Although there are *rpmA*, *rpmB*, …, *rpmJ* genes in *E. coli*, they are not paralogous. One particular read “AGTGCCGAGCCGAAGCATAAACAGCGCCAAGGCTGATTTTTTCGCATATT” occurs 2684 times in SRR1536586.sra. It matches perfectly to the 36 nt at the 3′ end of *rpmJ* and 14 nt immediately downstream. However, Cufflinks output reported a count of only 2114 reads for *rmpJ* instead of 6426 (and consequently the much reduced RPKM in Figure 4). I originally suspected that *rpmJ* may be in an operon with an immediate downstream gene so that some read overlapping *rpmJ* and the downstream gene would be divided between the two. However, the downstream gene, which is *rpsM*, is 146 nt away. It is difficult to reconcile 6426 nonambiguous read matches to Cufflinks’ 2114. Similarly, *rpmD* and *rplV* (Figure 4) has 14,468 and 22,747 unambiguous read matches, respectively, but the corresponding counts in Cufflinks output are only 8108 and 11,801, respectively. Note that *rpmD* and *rplV* are also single-copy genes with no ambiguous read matches. *E. coli* genes *rpmA*–*rpmJ* are not paralogous, neither are *rplA*–*rplY*.

**Figure 5:**
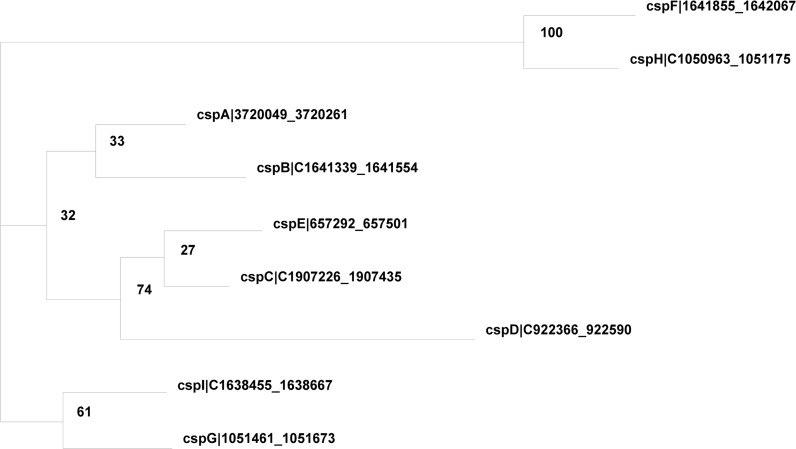
Phylogenetic relationship among paralogous genes *cspA* to *cspI* in *E. coli*, based on coding sequences, with bootstrap values next to internal nodes. Sequences were aligned by MAFFT (Katoh and Toh 2008) with accurate L_INS-i option and a maximum of 16 iterations. Coding sequences were first translated in amino acid sequences, which are aligned with BLOSUM62 matrix. Nucleotide sequences were then aligned against aligned amino acid sequences. Phylogenetic analysis was done with PhyML (Guindon *et al.* 2010). All these analyses were automated in DAMBE (Xia 2013, 2017).

The last outlying gene (*cpsA* in Figure 4) does involve a paralogous gene family (Figure 5). *cspA* has 19,776 unambiguous read matches, but Cufflinks output has only 10,957, which resulted in a much lower RPKM than that from ARSDA (Figure 4). Also puzzling are the counts involving *cspF* and *cspH*. There are 264 unambiguous read matches to *cspF* and 58 to *cspH*. There are also 55 reads that match well to both *cspF* and *cspH*, with 27 of them matching *cspF* better than *cspH*, and 28 matching *cspH* better than *cspF*. So we may assign (264 + 27) reads to *cspF* and (58 + 28) reads to *cspH*, with relative proportions of 0.7719 and 0.2281 for *cspF* and *cspH*, respectively. Twelve reads match *cspF* and *cspH* equally well (the same *e*-value and the same BitScore), so we assign them proportionally to the two genes, *i.e.*, 12*0.7719 to *cspF* and 12*0.2281 to *cspH*. The final counts for *cspF* and *cspH* are 300.2626 and 88.7374, respectively. However, Cufflinks output shows counts of 2 and 63 for *cspF* and *cspH*, respectively. The discrepancy is particularly striking given that gene expression from ARSDA and Cufflinks are mostly concordant (Figure 4). The alternative allocation to paralogous genes as specified in Equations (9) and (10) does not help reconcile the discrepancy. I hope that these numbers will prompt authors of Cufflinks to be more explicit about how they treats counts.

### Software and data availability

ARSDA is freely available at http://dambe.bio.uottawa.ca/ARSDA/ARSDA.aspx, together with a QuickStart.PDF file showing HTS file conversion from FASTA/FASTQ file to FASTA+ format, three types of HTS data quality visualization tools, and downstream characterization of gene expression. It is a Windows program, but can run on any computer with .NET framework installed (*e.g.*, Macintosh and Linux with MONO installed and activated). The BLAST databases derived from HTS reads for several model species, in which sequence IDs are in the format of SeqID_CopyNumber, are deposited at coevol.rdc.uottawa.ca. One can use these BLAST databases with ARSDA to characterize gene expression or other analysis. The QuickStart.PDF file available at the same site details the use of ARSDA, either alone or in conjunction with the free DAMBE software (Xia 2013, 2017). The source code is available as a zipped supplemental file ARSDA.Src.zip in https://github.com/xuhuaxia/ARSDA.

## Supplementary Material

Supplemental material is available online at www.g3journal.org/lookup/suppl/doi:10.1534/g3.117.300271/-/DC1.
